# Supplementary figures and images for: TMExplorer: A tumour microenvironment single-cell RNAseq database and search tool
Source: PLoS One. 2022 Sep 9;17(9):e0272302. doi: 10.1371/journal.pone.0272302 (PMC9462821; doi:10.1371/journal.pone.0272302)

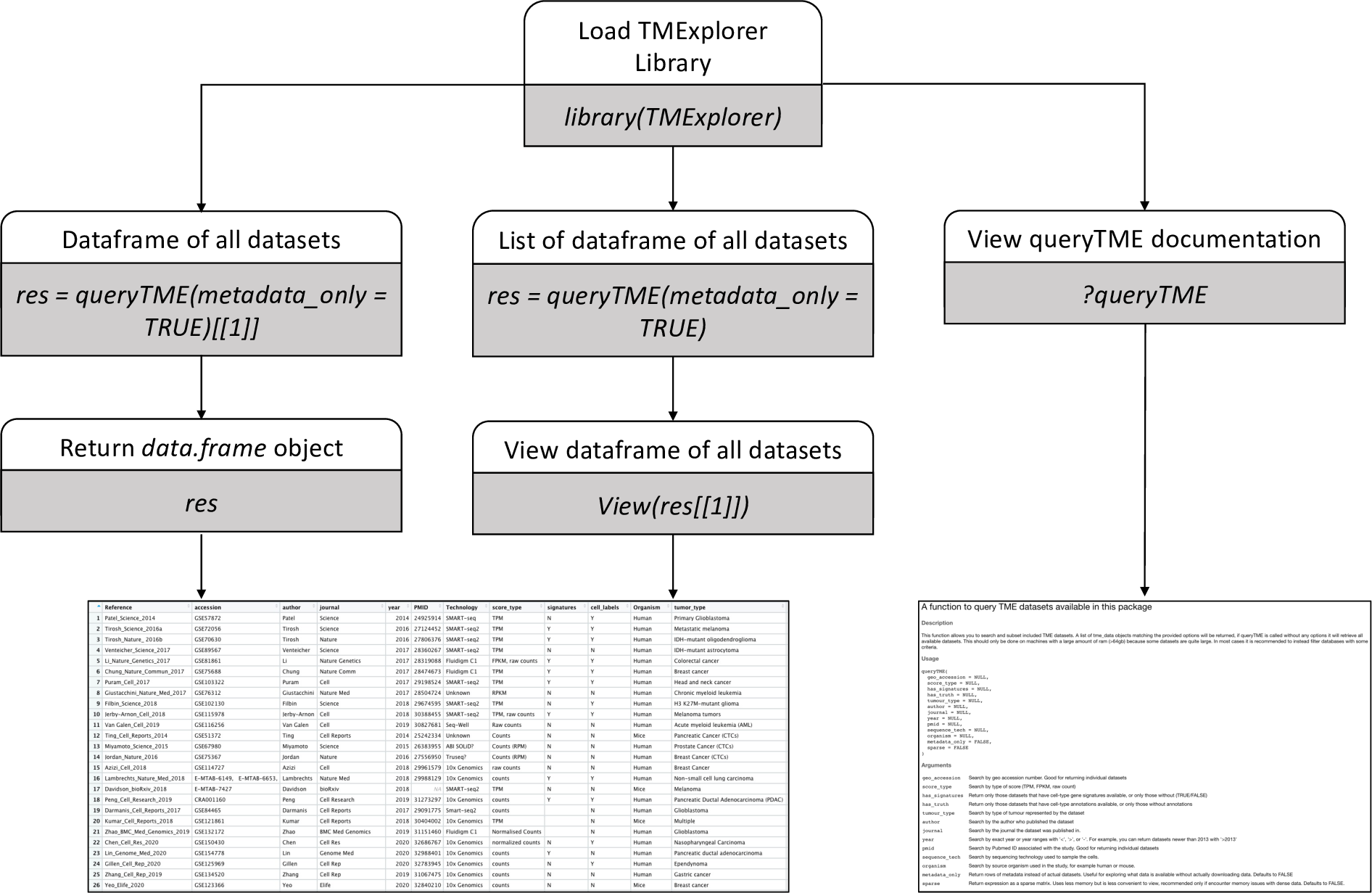

Supplement: S1 Fig — Users can view the TMExplorer database metadata of scRNA-seq datasets, interact with the metadata as a dataframe object, and view the TMExplorer documentation of function arguments included in the package. (TIF) [file pone.0272302.s001.tif]

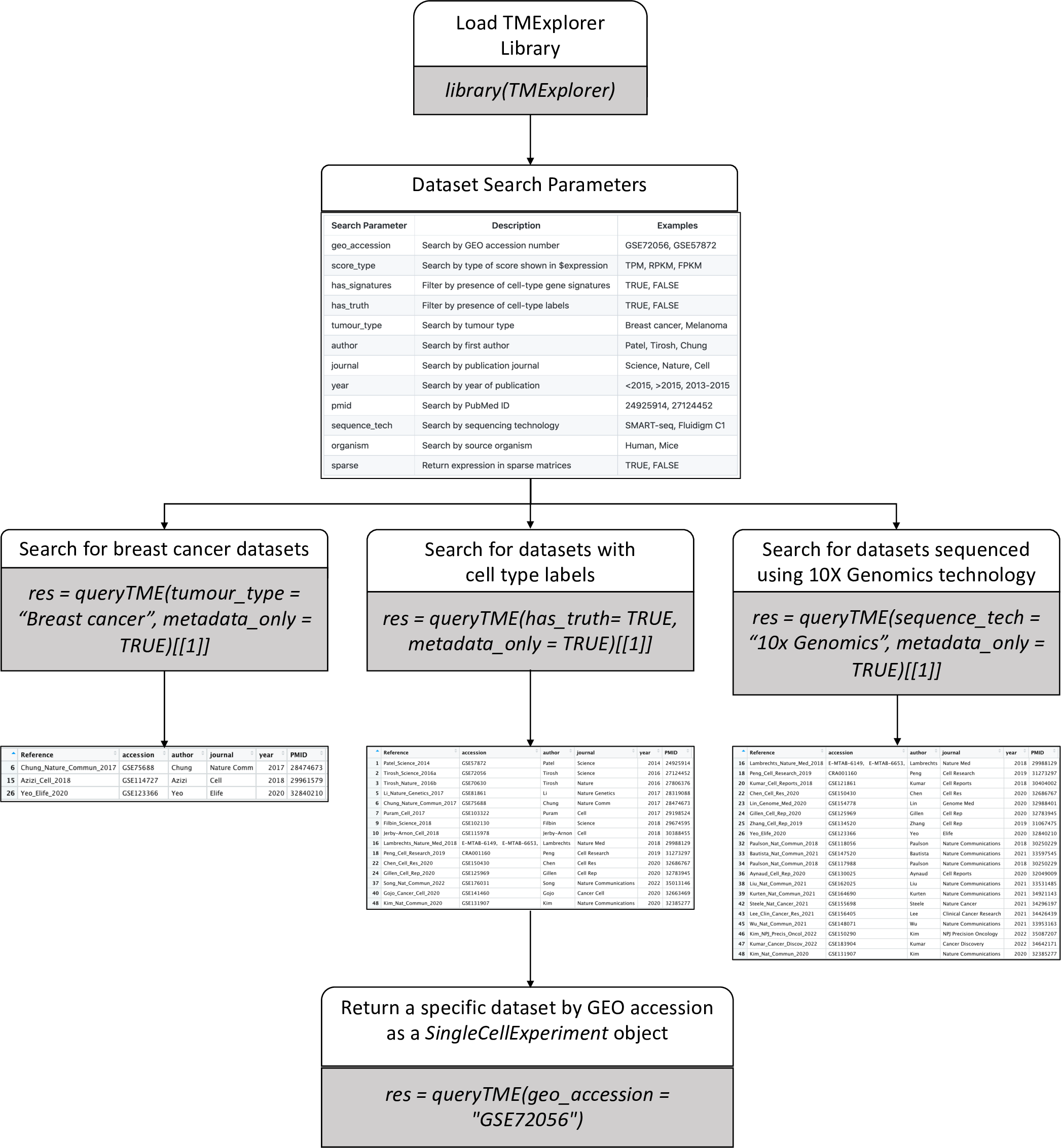

Supplement: S2 Fig — A set of searchable parameters can be used to filter scRNA-seq datasets. The users can search for specific datasets using user-specified parameters, and return one specific dataset as a SingleCellExperiment object for downstream analysis. (TIF) [file pone.0272302.s002.tif]
